# Innovative Use of Stone Waste for Sustainable Fertility Management in Tropical Soils

**DOI:** 10.1021/acsomega.6c00884

**Published:** 2026-05-19

**Authors:** Renata Coura Borges, Thiago de Sales Machado Freitas, Julia Oliveira Fernandes, Everaldo Zonta, Izabela Gouveia Nascimento, Fernando Henrique Cincotto, Marcos Gervasio Pereira, Luiz Alberto da Silva Rodrigues Pinto, Cassiano Augusto Rolim Bernardino

**Affiliations:** † Department of Soils, 67825 UFRRJ, Universidade Federal Rural do Rio de Janeiro, Seropédica 23897-000, Brazil; ‡ Department of Analytical Chemistry, Institute of Chemistry, 28125 Universidade Federal do Rio de Janeiro, Rio de Janeiro 21941-909, Brazil

## Abstract

The sustainable reuse of mining and stone-processing waste is increasingly recognized as a strategy for soil remineralization and circular resource management in agriculture. However, the geochemical interactions between ornamental stone waste and highly weathered tropical soils are still insufficiently understood. This study evaluated the effects of incorporating ornamental stone waste on the chemical, physical, and mineralogical properties of a Red-Yellow Argisol rich in organic matter. A greenhouse experiment was conducted using four treatments: control soil and soil amended with 5%, 10%, and 15% stone waste. The incorporation of stone waste significantly improved soil chemical properties, increasing pH from 4.3 (control) to 5.6, 6.3, and 6.9 in the 5%, 10%, and 15% treatments, respectively. Base saturation increased from 48% in the control soil to values above 90% in all amended treatments, while exchangeable acidity was substantially reduced. Calcium concentrations increased from 4.5 to 13.1 cmolc dm^–3^ and magnesium from 1.0 to 3.1 cmolc dm^–3^, indicating enhanced nutrient availability. Mineralogical analyses identified quartz, dolomite, feldspar, and enstatite as dominant phases, and their incorporation increased the relative abundance of silicates and carbonates in the soil. Fourier-transform infrared spectroscopy indicated the formation of organo-mineral associations, suggesting enhanced stabilization of soil organic matter. In terms of physical properties, bulk density increased from 0.73 to 0.93–1.03 g cm^–3^, while macroporosity decreased from 18% to 8–12%, and microporosity increased up to 35% depending on the treatment. These changes reflect structural modifications associated with the addition of fine mineral particles. Overall, the results demonstrate that ornamental stone waste acts as an effective soil remineralizer, improving soil fertility and altering structural properties, while providing a sustainable alternative for the reuse of mining byproducts in tropical agricultural systems.

## Graphical abstract

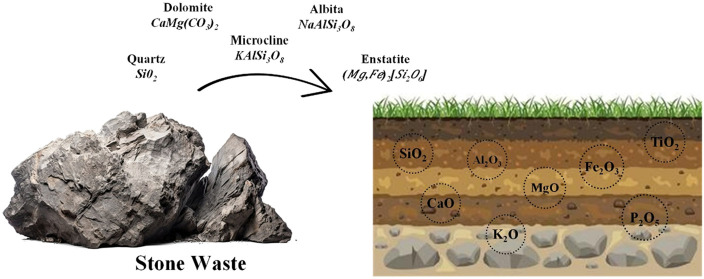

## Environmental Implication

The use of remineralizers represents a sustainable strategy for improving the fertility and resilience of tropical soils. By providing primary minerals that stimulate weathering and the gradual release of nutrients over the medium to long-term, these inputs promote root growth and the formation of stable aggregates, strengthening the structure and organic matter of the soil. In addition, they reduce dependence on chemical fertilizers, thereby decreasing environmental impacts. The use of stone waste, which is widely available in the region and low in cost, contributes to restoring soil health and the sustainability of Brazilian agricultural systems.

## 1 Introduction

Brazil is one of the world’s largest food producers, and the agribusiness sector accounts for approximately one-fifth of the national Gross Domestic Product when considering the entire production chain, including inputs, primary production, agro-industrial processing, and distribution.^1,2^ However, the conventional model of Brazilian agriculture is highly dependent on the import of soluble fertilizers, which accounts for approximately 80% of the country’s total fertilizer demand. This dependence varies among the main macronutrients: Brazil imports about 95% of its potassium (K) fertilizers, 70–75% of nitrogen (N), and 50–60% of phosphorus (P) used in agricultural production. This reliance increases production costs, reduces competitiveness, and heightens vulnerability to international market fluctuations, while reinforcing dependence on a limited number of input-supplying countries.^3,4^

Soil fertility in highly weathered tropical soils is often limited by low nutrient availability, intense leaching, and the progressive depletion of essential mineral elements. In these environments, soil remineralization using finely ground rocks has been increasingly investigated as an alternative strategy to improve nutrient supply and enhance long-term soil fertility. Rock powders may gradually release macro- and micronutrients through weathering processes, contributing to the replenishment of elements removed by crop uptake and erosion.^3,5^

In recent years, the agricultural use of mining and quarrying byproducts has attracted attention as part of sustainable soil management strategies. In addition to providing nutrients, these materials may influence soil chemical equilibria, mineral transformations, and interactions with soil organic matter. Such processes can affect important properties such as cation exchange capacity, pH buffering, and the formation of organo-mineral associations that contribute to soil structural stability.^5^

Among these materials, residues generated during the processing of ornamental stones represent a potentially valuable but still underexplored resource. The ornamental stone industry generates significant quantities of fine waste during cutting and polishing operations. These stone waste commonly contain minerals such as quartz, feldspars, carbonates, and mafic silicates, which may participate in geochemical reactions when incorporated into soils.^6–8^ The reuse of these materials in agriculture may therefore represent both an alternative source of nutrients and a strategy to reduce the environmental burden associated with industrial waste disposal, while contributing to circular-economy approaches in the mining sector.^6,7^

Although rock powders have been reported as remineralizers, most studies focus on single rock types or their role as slow-release nutrient sources. The novelty of this study lies in evaluating ornamental stone processing waste as a multifunctional remineralization material, combining mineralogical, chemical, and physical analyses to assess its effects on soil fertility and structure in a tropical Argisol with high organic matter content. In addition, this work explores the potential of an abundant industrial residue from the ornamental stone sector as a circular-economy strategy for sustainable soil management.^6,7^

The assessment of land suitability and capability is a fundamental component of sustainable agricultural management, as it provides a basis for selecting appropriate soil management strategies and optimizing land use according to inherent soil conditions.^9^ This approach is particularly relevant when evaluating the application of remineralizers, such as ornamental stone waste, since the effectiveness of mineral amendments is strongly dependent on the initial soil properties and its capacity to respond to nutrient inputs. In this context, integrating soil suitability assessment with detailed mineralogical and geochemical analyses is essential to comprehensively evaluate soil fertility restoration processes and ensure long-term sustainability.

Based on this framework, we hypothesize that the addition of ornamental stone waste, due to its silicate- and carbonate-rich mineralogical composition, promotes beneficial geochemical interactions that increase soil pH, cation exchange capacity, and base saturation, while also modifying soil physical structure and enhancing organo-mineral associations. These processes are expected to improve nutrient availability and contribute to the gradual recovery of soil functionality in degraded environments.

Therefore, this study aimed to evaluate the effects of ornamental stone waste incorporation on the chemical, physical, and mineralogical properties of a Red-Yellow Argisol with high organic matter content, considering its potential as an alternative remineralizing material within the Brazilian regulatory framework for agricultural remineralizers..^10,11^

## 2 Material and Methods

### 2.1 Constitution and Characteristics of the Stone Waste

The stone waste used in the experiment was produced in the state of Rio de Janeiro during the rock polishing stage at a marble workshop, whose geographic coordinates are 22° 54′ 26.89″S and 43° 16′ 11.91″W. The samples were collected from eight different points in the stone waste pile at the marble workshop, according to the Brazilian standard NRB-ABNT 10007, “Sampling by waste procedure”.^12^ These samples were air-dried, crushed, and sieved to <2 mm. Afterward, the samples were weighed and subjected to several quantitative and qualitative analyses.

The collected waste comes from the cutting and polishing of blocks, which is carried out using cutting was performed with diamond blades/discs, and polishing with ceramic discs.^13^ According to Karaca et al.,^14^ stone waste can be classified into three groups: solid, powder, and semislurry. Based on the characteristics of the waste used in this study, it was classified as semislurry waste. This waste comes from different types of ornamental rocks and is composed mainly of 50% granite and gneiss, 25% marble, and 25% quartzite.

Granite is an igneous rock composed of quartz, feldspar, micas, amphiboles, and pyroxenes. Its composition varies according to the formation process it undergoes. Owing to its mineralogy, granite processing waste may contain significant amounts of certain chemical elements considered macro- and micronutrients for plants. Among the macronutrients, potassium (K), calcium (Ca), and magnesium (Mg) are of particular interest. Iron (Fe) is among micronutrients, as it is present in the waste due to wear of the steel and iron tools (discs and blades) during the cutting and polishing of stones such as marble, granite, and quartzite. Small iron particles released from this wear become incorporated into the waste.

Gneiss primarily results from the metamorphism of granite. It is one of the oldest rocks in the world. Gneiss is a rock with significant mineralogical variation and metamorphic grade.^15^ During its formation, mineral reorientation occurs. This rock is composed of quartz (40–50%), greenish clinoamphibole (20–25%), epidote (7–10%), diopside (5–10%), plagioclase (5–8%), microcline (3–5%), and titanite (3–5%), with clinozoisite and carbonate (5–10%) also appearing as common accessories.^16^

Marble is a metamorphic rock derived from limestone. This type of rock contains more than 50% calcite and/or dolomite, that is, they are formed by the metamorphism of sedimentary rocks composed mainly of calcite and dolomites. It has a massive structure and a varied grain size.^17^ Marble composed primarily of calcite (CaCO_3_) and dolomite (CaMg­(CO_3_)_2_) is typically white; however, it appears in different colors due to the inclusion of other minerals: tremolite and diopside result in green, phlogopite, muscovite, and siderite in brown, magnesite and manganese oxides in pink, and graphite in black.^18^

This stone waste is classified as a remineralizer (RM). Stone waste is a byproduct/residue from mining. According to Law No. 12,890 (December 10, 2013), remineralizers are mineral materials processed mechanically to reduce particle size, capable of altering soil fertility by supplying macro- and micronutrients and improving soil properties.^19,20^ According to Brazilian legislation, it belongs to class “E,” meaning its raw material is exclusively of mineral origin, and for its application in agriculture, it must comply with the criteria established by Normative Instruction No. 05 of March 10, 2016.^11^ Thereafter, the material may be used, provided it meets the requirements for registration with the Ministry of Agriculture.

### 2.2 Experimental Design

The experiment was conducted in a greenhouse at the Geotechnics Department of the Federal University of Rio de Janeiro (UFRJ), located on Fundão Island in the northern zone of the city of Rio de Janeiro, located at approximately 22°50′S, 43°13′W. The soil studied was collected on Catalão Island and is classified as a Red-Yellow Argisol according to the Brazilian Soil Classification System (SiBCS), corresponding to a Haplic Lixisol in the FAO/WRB classification system.,^21^ with predominantly undulating relief. This soil was chosen because it is considered a soil with minimal anthropogenic disturbance. The collected soil samples were oven-dried at 40 °C for 72 h, or until constant weight was achieved, to avoid possible alterations in the mineralogical composition and in the stability of soil organic matter that may occur at higher temperatures, crushed, sieved through a 2000 μm mesh, and sent for particle size physical analysis.^22^ The experiments were conducted in a controlled environment. The test was set up in a completely randomized design, consisting of four treatments with three replicates per treatment, in which increasing amounts of stone waste were applied, and each treatment had three repetitions. The treatments were:

- Treatment 1:4000 g of soil
- Treatment 2:3800 g of soil + 200 g of stone waste
- Treatment 3:3600 g of soil + 400 g of stone waste
- Treatment 4:3400 g of soil + 600 g of stone waste

Application rates (5%, 10%, and 15% w/w) were defined based on previous studies on rock-derived amendments, which have demonstrated their capacity to enhance soil fertility through mineral weathering and nutrient release. These rates establish a controlled gradient to assess dose-dependent effects while ensuring agronomic relevance and environmental safety.^23,24^

### 2.3 Evaluation of the Chemical and Physical Properties of Stone Waste, Soil from Catalão, and the Treatments Applied

Soil fertility assessment under degradation conditions is a critical step in evaluating the effectiveness of soil remineralization strategies. Recent studies have demonstrated that integrating geochemical and soil property analyses provides a robust framework for understanding fertility dynamics and guiding soil management practices.^25^ In this context, the application of mineral inputs, such as stone waste, can contribute to the gradual restoration of soil fertility by enhancing nutrient availability and improving soil chemical attributes.

Furthermore, soil fertility evaluation frameworks benefit from integrated analytical approaches that combine different scales and techniques to better capture soil transformations.^26^ In the present study, this integrated framework is applied through laboratory-based chemical (ICP-OES) and mineralogical analyses to investigate the effects of stone waste application at different doses (T2–T4).

The chemical characterization of the stone waste, soil and treatments were performed according to standard soil fertility analysis procedures established by the Embrapa (2014).^27^ The analyses included soil pH (in water), exchangeable cations (Ca^2+^, Mg^2+^, K^+^, and Na^+^), exchangeable acidity (Al^3+^), potential acidity (H^+^+Al^3+^), cation exchange capacity (CEC), and base saturation (V%). These parameters were determined using routine methods such as extraction with appropriate solutions followed by titration or spectrometric determination, according to standardized protocols. The physical characterization was conducted through particle size distribution (granulometric analysis), to determine the proportions of different size fractions, following conventional procedures for mineral materials.

The chemical properties of the samples were characterized according to the methodology recommended by Embrapa (3ed., 2014),^27^ as described below. The Hydrogen Potential (pH) was measured using a combined electrode immersed in a solid-to-liquid (H_2_O) suspension at a ratio of 1:2.5 (10 g of soil to 25 mL of solution). A potentiometer (Orion model 710A, Orion Research Inc., Boston, MA, USA) was used for measurement. The pH of KCl was also determined using the same electrode and equipment, but with a 1:2.5 ratio of 1 mol L^–1^ KCl solution. The δpH was determined using the following equation:

$$\Delta \mathrm{pH}=\mathrm{pHKCl}-\mathrm{pH}H_{2}O$$

Where: ΔpH = delta pH; pHKCl = soil pH measured in 1 mol L^–1^ KCl solution; pHH_2_O = soil pH measured in distilled water.

Cation exchange capacity (CEC): The cation exchange capacity was determined by extracting exchangeable calcium (Ca^2+^) and magnesium (Mg^2+^) with a KCl solution (1 mol L^–1^) and sodium (Na^+^) and potassium (K^+^) with a Mehlich-1 extractant solution. The Ca^2+^ and Mg^2+^ ions were then measured by Inductively Coupled Plasma Optical Emission Spectrometry-ICP-OES (ULTIMA 2 Horiba Inc., Japan), while Na^+^ and K^+^ were measured using a Flame Photometer (DM-6, Digimed). The exchangeable aluminum content (Al^3+^) was obtained by extraction with KCl solution (1 mol L^–1^) and determined using the volumetric titration with standardized NaOH solution (0.025 mol L^–1^). Potential acidity (H^+^ + Al^3+^) was determined by extraction with an extraction with 1 mol L^–1^ calcium acetate solution (pH 7.0), titrated with NaOH using phenolphthalein indicator.

Analytical instruments, including ICP-OES (ULTIMA 2, Horiba) and the flame photometer (DM-6, Digimed), were calibrated using appropriate standard solutions, with periodic verification of instrumental stability and linearity. Additionally, analytical blanks, certified reference materials, Montana Soil (NIST 2710) and San Joaquin Soil (NIST 2709), as well as triplicate analyses were included to assess elemental concentrations and to ensure analytical precision and reproducibility. The accuracy of the analytical methods was evaluated through percent recovery of the certified reference materials, with recovery values ranging between 90% and 110% for the analyzed elements.

To determine the organic carbon, the organic matter was oxidized with potassium dichromate in concentrated H_2_SO_4_, where the acid provides the heat of reaction. The excess dichromate was titrated with ammonium ferrous sulfate ferrous ammonium sulfate (Mohr’s salt). The organic matter was determined gravimetrically by loss-on-ignition, based on mass loss after combustion at high temperature and, consequently, the loss of soil mass from the elimination or mixture subjected to high temperature for a certain period. To determine the organic matter (OM), the difference between the initial weight samples oven-dried at 105 °C and the weight calculated after incinerated at 550–600 °C.^28^ Particle size analysis was performed to determine the particle size of the stone waste, soil, and treatments applied using the method suggested by Ziervogel and Bohling.^29^ Four grams of each sample were initially treated with hydrogen peroxide (H_2_O_2_, 30%) to remove organic matter. The reagent was added gradually on a daily basis to control the reaction and avoid excessive effervescence, until complete oxidation of organic matter was achieved, not exceeding a period of 14 days. After the reaction ceased, the samples were repeatedly washed with distilled water to remove residual reagents and reaction byproducts. The solid phase was then separated from the supernatant by centrifugation at 3000 rpm (≈1000 g) for 5 min, and this washing–centrifugation cycle was repeated until neutral pH was reached.

Subsequently, the samples were dispersed using 4.5 mol L^–1^ sodium hexametaphosphate solution, acting as a chemical dispersant to prevent particle flocculation. The suspension was mechanically agitated for 24 h to ensure complete dispersion of the particles. Prior to analysis, the samples were homogenized and, when necessary, subjected to ultrasonic treatment to further enhance dispersion.

Particle size distribution was determined using a laser diffraction particle size analyzer (CILAS 1064), based on the Mie scattering theory. The measurements were performed under controlled conditions, with appropriate refractive index parameters for the solid phase and dispersant medium. Each sample was analyzed at least in duplicate to ensure reproducibility.

### 2.4 Fluorescence Analysis and X-ray Diffractometry

The X-ray diffraction (XRD) was used to identify and quantify crystalline phases of each sample. The samples were prepared for EDXRF analysis following standard procedures to ensure homogeneity and analytical accuracy. Initially, the samples were dried at low temperature (≈ 40 °C) to avoid mineralogical alterations and then finely ball mill until reaching a particle size of <63 μm. This step was essential to obtain a uniform and representative powder, minimizing particle size effects on X-ray fluorescence measurements.

After grinding, the samples were homogenized and stored in clean, contamination-free containers. For analysis, an appropriate amount of the powdered sample was placed in sample holders (cups) equipped with a thin X-ray transparent film (e.g., Mylar or polypropylene). The material was carefully leveled and lightly pressed to obtain a flat and uniform surface, ensuring consistent interaction with the incident X-ray beam.

This analysis was Department of Geochemistry, Fluminense Federal University (UFF). X-ray fluorescence (XRF) was used to determine major and minor element composition of the stone waste, allowing the identification of its major and minor constituents. The energy-dispersive X-ray fluorescence (EDXRF) equipment used was a EDXRF (Shimadzu EDX-800HS, Rh source, Si­(Li) detector cooled with liquid nitrogen). For the investigation of heavy elements should be Ti to U, a voltage of 50 keV and a current of 33 μA were applied. For light elements C to Sc, 15 keV and 297 μA, with an acquisition time of 100 s and a 10 mm collimator. The analyses were performed at the X-ray Laboratory of the Institute of Nuclear Engineering, located at the University City, Fundão Island, Rio de Janeiro, Brazil.

### 2.5 Scanning Electron Microscopy

Morphological characterization of soil, stone waste, and treated samples was performed using scanning electron microscopy (SEM) at the Multiuser Ultrastructure Laboratory, Federal Rural University of Rio de Janeiro (UFRRJ), Seropédica, Brazil. Micrographs were obtained using a Phenom ProX SEM (Thermo Fisher Scientific) equipped with an energy-dispersive X-ray spectroscopy (EDS) detector for elemental analysis.

Samples were air-dried at room temperature and gently disaggregated using an agate mortar and pestle to preserve structural features. The material was subsequently sieved (<2 mm) to obtain a representative and homogeneous fraction. For SEM analysis, small aliquots were mounted on aluminum stubs using double-sided carbon adhesive tape. Prior to imaging, samples were cleaned with a nitrogen jet to remove loose particles.

Analyses were conducted under high vacuum conditions at an accelerating voltage of 15 kV. Representative micrographs were acquired at different magnifications to evaluate particle morphology and organo-mineral associations.

### 2.6 Fourier Transform Infrared (FTIR) Spectroscopy

The FTIR spectra were obtained in the range of 4000–400 cm^–1^, using the Attenuated Total Reflectance ATR mode, FTIR spectra were recorded using a Bruker VERTEX 70 spectrometer with OPUS v6.5 software.

### 2.7 Statistical Analysis

Multivariate statistical analysis was applied to evaluate differences among treatments. Principal component analysis (PCA) was performed using STATISTICA v7 (StatSoft, Tulsa, USA). PCA allows the variability of the data to be summarized into a few principal components, which explain most of the total variance. PCA provides factor loadings that indicate the weight of each variable in the component. PCA factor loadings identified variables most responsible for differentiating treatments.

In addition, Pearson’s correlation analysis was performed to assess the strength and direction of linear relationships among the evaluated physicochemical soil properties. The Pearson correlation coefficients (r) were calculated, and their significance was tested at the 5% and 1% probability levels. This analysis enabled the identification of significant associations between variables, supporting the interpretation of the multivariate results.

## 3 Results and Discussion

### 3.1 Chemical Characterization and Standardization

XRF was used to identify and quantify the elemental composition of rock powder, Catalão soil, and treatments.^30^ The XRF results are shown in Table 1. The main components of the rock powder were silicon dioxide (SiO_2_), calcium oxide (CaO), and aluminum oxide (Al_2_O_3_). Catalão soil was dominated by SiO_2_, with Al_2_O_3_ and Fe_2_O_3_ as secondary components. In contrast, the application of increasing doses of rock powder resulted in different responses regarding the three primary elements. Treatment T2 showed an oxide composition similar to the soil, while T3 and T4 shifted toward the residue profile, with SiO_2_ dominant, followed by Al_2_O_3_ and CaO, where CaO exceeded Al_2_O_3_. The results also revealed variations were observed in K_2_O, MgO, Fe_2_O_3_, and Na_2_O concentrations among residue, soil, and treatments. Minor elements detected were Ti, P, S, Ba, Mn, Cu, Sr, Zr, Rb, and Zn. Differences in minor oxide concentrations were minimal, indicating similar compositions across sources.

**Table 1:** Chemical Elements Obtained on the X-ray Fluorescence

elements (%)	stone waste	T1	T2	T3	T4	granite^a^	marble^b^
SiO_2_	49.680	59.919	64.029	64.235	63.524	61.40	1.39
CaO	19.743	3.359	3.796	4.008	6.269	6.30	54.5
Al_2_O_3_	11.052	18.745	22.098	21.845	19.559	3.69	0.32
K_2_O	4.104	6.065	3.789	3.737	3.821	3.75	<0.06
MgO	3.406					1.70	0.64
Fe_2_O_3_	7.338	6.354	3.971	3.819	4.343	3.66	0.14
TiO_2_	1.478	1.973	0.909	1.136	1.118		
P_2_O_5_	0.939	0.928	0.367	0.381	0.103		
SO_3_	1.207	1.595	0.121	0.371	0.381	0.05	<0.10
BaO		0.524	0.699	0.309	0.593		
MnO	0.127	0.092		0.049	0.057		
CuO	0.038	0.015	0.010	0.014	0.012		
SrO	0.090	0.148	0.082	0.084	0.092		
ZrO_2_	0.062	0.232	0.092	0.095	0.100		
Rb_2_O	0.021	0.023	0.013	0.014	0.014		
ZnO	0.020	0.028	0.014	0.016	0.015		

a Abukersh and Fairfield, 2011.

b Rodrigues et al., 2015.

Chemically, the comparison of stone waste, soil, and treatments with pure granite and marble waste shows marked differences in constituent concentrations. Abukersh and Fairfield^31^ reported the recycling of red granite and found SiO_2_ (61.40%), CaO (6.30%), and K_2_O (3.75%) as the three main elements. On the other hand, Rodrigues et al.,^32^ determined the mechanical and chemical properties of marble waste and found values of CaO (54.50%), SiO_2_ (1.39%), and MgO (0.64%). The stone waste averaged SiO_2_ (54.90%), CaO (15.94%), and Al_2_O_3_ (13.3%). These results show that the chemical composition of waste generated in the ornamental stone industry differs. These differences reflect geological variability and rock diversity among producing regions. Therefore, the use of stone waste does not pose a significant risk of environmental contamination by these elements. According to Normative Instruction no. 5 from MAPA, remineralizers must meet minimum standards for agricultural use:

- granulometry material must have 100% passing 2.0 mm and at least 70% passing 0.3 mm;
- total base sum (CaO + MgO + K_2_O) of at least 9%;
- total K_2_O content of at least 1%;
- maximum free silica content between 18% and 25%;
- phosphorus content (P_2_O_5_) above 1%.

According to the results in Table 1, all criteria were met by stone waste, although silica content (∼49%) exceeded the regulatory limit of 18–25%. The silica content in the studied material is slightly above the reference value considered in the regulatory framework for remineralizers. However, this does not prevent its agricultural use as a soil remineralizer. Silicon is widely recognized for its beneficial effects on plant growth and resistance to biotic and abiotic stresses. Thus, the observed silica content is not a restriction but a characteristic that may contribute positively to soil–plant interactions. The limits for micronutrients are presented in Table 2. The stone waste does not contain micronutrient levels exceeding the minimum concentrations established by MAPA, thus meeting requirements except for silica content.

**Table 2:** Minimum Contents of the Macronutrient Phosphorus and Micronutrients in Remineralizers^a^ ^,^ ^b^

características	Teor total mínimo (% peso/peso)
Fósforo (P_2_O_5_)	1.0
Boro (B)	0.03
Cloro (Cl)	0.1
Cobalto (Co)	0.005
Cobre (Cu)	0.5
Manganês (Mn)	0.1
Molibdênio (Mo)	0.005
Níquel (Ni)	0.005
Zinco (Zn)	0.1

a Characteristics—minimum total content (% w/w).

b Fonte^10^: Brasil, 2016.

Remineralizers are ground rocks, and their degree of fragmentation influences material reactivity. Smaller particle sizes increase surface area and enhance soil reactivity. IN N° 5 presents the particle size distribution of the remineralizers. In this study, particle size distribution was used to determine the surface area for chemical and physical reactions and was regrouped into four classes for comparative analysis (Table 3): clay (≤2 μm), silt (2–63 μm), fine sand (63 to 600 μm), and coarse sand (600 to 2000 μm). Silt dominated the stone waste sample (71%), indicating that stone waste can be described as fine dust with high specific surface area. Clay comprised approximately 15% of the residue.

**Table 3:** Mean Fertility Concentration and Particle Size Analysis of the Stone Waste^a^

	pH_H_2_O_	pH_KCl_	P	K	K^+^	Ca^2+^	Mg^2+^	Na^+^	Al^3+^	H^+^ + Al^3+^	V	MO	Corg
	1:2.5	1:2.5	mg L^–1^		cmol_c_·dm^–3^						%		
Stone Waste	8.9	8.4	1.0	47	0.03	4.5	1.0	0.3	0.00	0.00	100	0.00	0.00
Catalão Soil (T1)	4.3	3.2	657	27	0.07	4.99	1.9	0.8	0.52	8.22	48	92.2	53.59
T2	5,6	5.1	838	27	0.07	12.9	2.2	1.0	0.00	2.08	90	75.9	44.12
T3	6.3	5.9	872	33	0.08	11.1	3.1	1.0	0.00	2.28	90	78.9	45.90
T4	6.9	6.0	669	24	0.06	13.1	2.1	0.9	0.00	1.17	93	75.8	44.12

	clay (%)	silt (%)	thin sand (%)	thick sand (%)
Stone Waste	15	71	11	3
Catalão Soil	2	15.3	47.7	35
T2	10	18	45	27
T3	10	26	44	20
T4	10	29	43	18

a pH in water; P,K: Mehlich Extractor1; Ca, Mg and Al: KCl Extractor 1N; V: alkaline elements saturation index; m: Aluminum saturation index; particle size analysis on laser analyzer model 1064 Cilas.

Granulometry is a crucial factor influencing nutrient availability from remineralization. Fine fractions such as clay and silt release nutrients rapidly through weathering, whereas coarse fractions release nutrients gradually. Thus, adding stone waste can contribute to the incorporation of new clay minerals and increase cation exchange capacity. Catalão soil was sandy, but treatments T2–T4 showed increased clay and silt fractions (18–29%), proportional to the applied dose, compared to the control with clay and silt contents of 18, 26, and 29%, respectively. This approach is economically and ecologically viable, relying on rock type and particle size distribution in the soil.^33^

### 3.2 Evaluation of the Agricultural Use of Stone Waste

Macronutrients (C, O, H, N, P, S) and essential mineral nutrients (K, Ca, Mg, Fe, Mn, Mo, Cu, B, Zn, Cl, Na, V, Co, Si) were considered. Table 2 shows pH variation ranging from 8.9 in rock powder to 4.3 in soil, with T2–T4 increasing to 5.6, 6.3, and 6.9, respectively, indicating considerable variability. According to the Fertilization and Liming Manual, soils with pH > 6.0 generally show high base saturation (>80%), enhancing retention of cations such as K, Ca, and Mg. The pH influences the solubility of nutrients in the soil and their absorption by plants, with Embrapa^27^ indicating optimal pH (5.5–6.5) was achieved in T2 and T3. Soils with V% greater than 50% are classified as eutrophic.^34–36^ Based on this, stone waste increased V% across all treatments.

As Tables 4 and 5 show, the range of interpretation for the evaluated parameters was based on the Fertilization and Liming Manual developed by the Brazilian Society of Soil Science.^37^

**Table 4:** Interpretation Ranges for Phosphorus (P), Potassium (K) and pH Levels^a^

P (mg dm^–3^)	K (mg dm^–3^)	pH	interpretation
≤7.0	≤20.0	≤5.0	Very low
7.1–14.0	21.0–40.0	5.1–5.4	Low
14.1–21.0	41.0–60.0	5.5–6.0	Medium
21.0–42.0	61.0–120.0	≥6.0	High
>42.0	>120.0		Very high

a Reference 34 SBCS, 2004.

**Table 5:** Interpretation Ranges for Calcium (Ca), Magnesium (Mg) and Organic Matter (OM) Contents^a^

Ca (cmol_c_ dm^–3^)	Mg (cmol_c_ dm^–3^)	MO (%)	interpretação
≤2.0	≤0.5	≤2.5	Low
2.1–4.0	0.6–1.0	2.6–5.0	Medium
>4.0	>1.0	>5.0	High

a Reference 34 SBCS, 2004.

In soils of tropical regions, where soils dominated by 1:1 clays and Fe/Al oxides, which have low CEC, the management of residues with high pH can be relevant, as it promotes an increase in the soil CEC. This occurs because, higher pH increases pH-dependent negative charges on soil minerals. Furthermore, pH plays a fundamental role in controlling the bioavailability of trace elements, radionuclides, and rare earth elements (REEs) in soils, as well as in solubility/precipitation and complexation reactions. The use of stone waste with high pH enhances CEC and nutrient bioavailability.^33^

Regarding the available P content, the stone waste contained very low P (1.0 mg dm^–3^), according to the Fertilization and Liming Manual.^34^ In contrast, the soil from Catalão soil had very high P (657 mg dm^–3^); T2, T3, and T4 increased to 838, 872, and 669 mg dm^–3^, respectively, which are considered very high. These results indicate that T2 and T3 had higher levels of available phosphorus, linked to optimal pH (5.5–6.5), while reduced phosphorus in T4 may reflect precipitation with Al and Fe at pH above 6.5. As pH increases, soluble phosphate ions (H_2_PO_4_^–^ and HPO_4_^2–^) react with Fe^3+^ and Al^3+^ to form insoluble or poorly soluble compounds (e.g., Fe- and Al-phosphates). These precipitated forms are less available for plant uptake, reducing the concentration of available phosphorus in the soil solution.

Available potassium ranged from medium to low, with 47 mg dm^–3^ in stone waste and 24 mg dm^–3^ in T4. In soils, exchangeable potassium can rapidly convert to nonexchangeable forms, increasing leaching risk and losses due to the soil’s tendency to reach equilibrium.^35^ This highlights the importance of adequate nutrient management to prevent the depletion of natural soil reserves.^36^ Furthermore, K is essential for photosynthesis, translocation, and ionic balance.^37^

Stone waste contained no organic matter, while Catalão soil had high organic matter (92.2 g kg^–1^), classified as high according to the Fertilization and Liming Manual.^34^ Organic matter decreased to 75.9–78.9 g kg^–1^ in T2–T4, which were still classified as high. Therefore, it can be inferred that OM content was reduced following the application of the treatments. This reduction may reflect complexation with clays and minerals in stone waste. present in the stone waste. The decrease is primarily attributed to dilution from adding mineral material to the soil matrix, rather than decomposition or loss of native organic matter. The incorporation of the rock-derived material increases the mineral fraction of the soil, which proportionally reduces the measured organic matter content when expressed on a mass basis. There is no evidence that the stone waste promoted accelerated mineralization or degradation of the existing soil organic matter.

Calcium increased from 4.5 to 13.1 cmolc dm^–3^, and magnesium from 1.0 to 3.1 cmolc dm^–3^. The Ca:Mg ratio influences nutrient competition and plant development.^38^ Salvador et al.,^39^ in an experimental study on the effects of different Ca:Mg ratios, reported that a 3:1 ratio in the soil solution is optimal for maintaining the balance of Ca, Mg, and K in the foliar content of soybean. The Ca:Mg ratios exceeded the optimal 3:1, except in Catalão soil, which had 2.5:1.

Brazilian latosols and argisols are highly weathered, acidic, and nutrient-poor, dominated by Fe and Al oxides. These soils are typically acidic and poor in mineral nutrients. Consequently, stone waste improved pH, cation exchange capacity, and base saturation, while reducing exchangeable acidity. The increase in CEC was due to the formation of new clay minerals during residue weathering, as well as an increase in pH, which enhances the negative charge on the surface of colloids that are often pH-dependent. The use of stone waste to produce agricultural fertilizers, as well as in the process of restoring acidic and low-fertility soils, has had a positive impact on improving certain chemical properties of the soil, resulting in an increase in pH, CEC, and base saturation (V%). There was also a marked reduction in the exchangeable acidity.^40–42^ However, OM and organic C decreased, likely due to pH changes. Based on these results, applying stone waste to Catalão soil corrected pH, enhanced nutrient bioavailability, stimulated soil biota, and improved erosion control, supporting better plant development.^43^ In addition, applying stone waste may reduce reliance on synthetic inputs, lowering energy and resource demands.

### 3.3 X-ray Diffractometry

The main crystalline phase components of the stone waste, as determined by X-ray diffraction, were quartz (SiO_2_), dolomite, feldspar, and enstatite (Figure 1). Dolomite provides calcium and magnesium and raises soil pH. From a sustainable development perspective, using stone waste reduces the impacts of limestone extraction and enables new use for this residue. Saygili et al.,^44^ Ibrahim et al.;^45^ Ogila et al.;^46^ Pastor et al.,^47^ Sabat and Muni;^48^ Igwe and Adepehin,^49^ and Sivrikaya et al.,^50^ have demonstrated that the stone waste has been shown to stabilize clay soils, reduce water absorption and improving geotechnical properties. XRD showed a dominant quartz peak at 26.6° 2θ, with secondary peaks at 20–21° and 50–52°, reinforcing this dominance, as described by Surendar et al.,^51^ for the Pachamalai samples, where quartz was the main mineral identified in the XRD and FTIR analyses.

**Figure 1:**
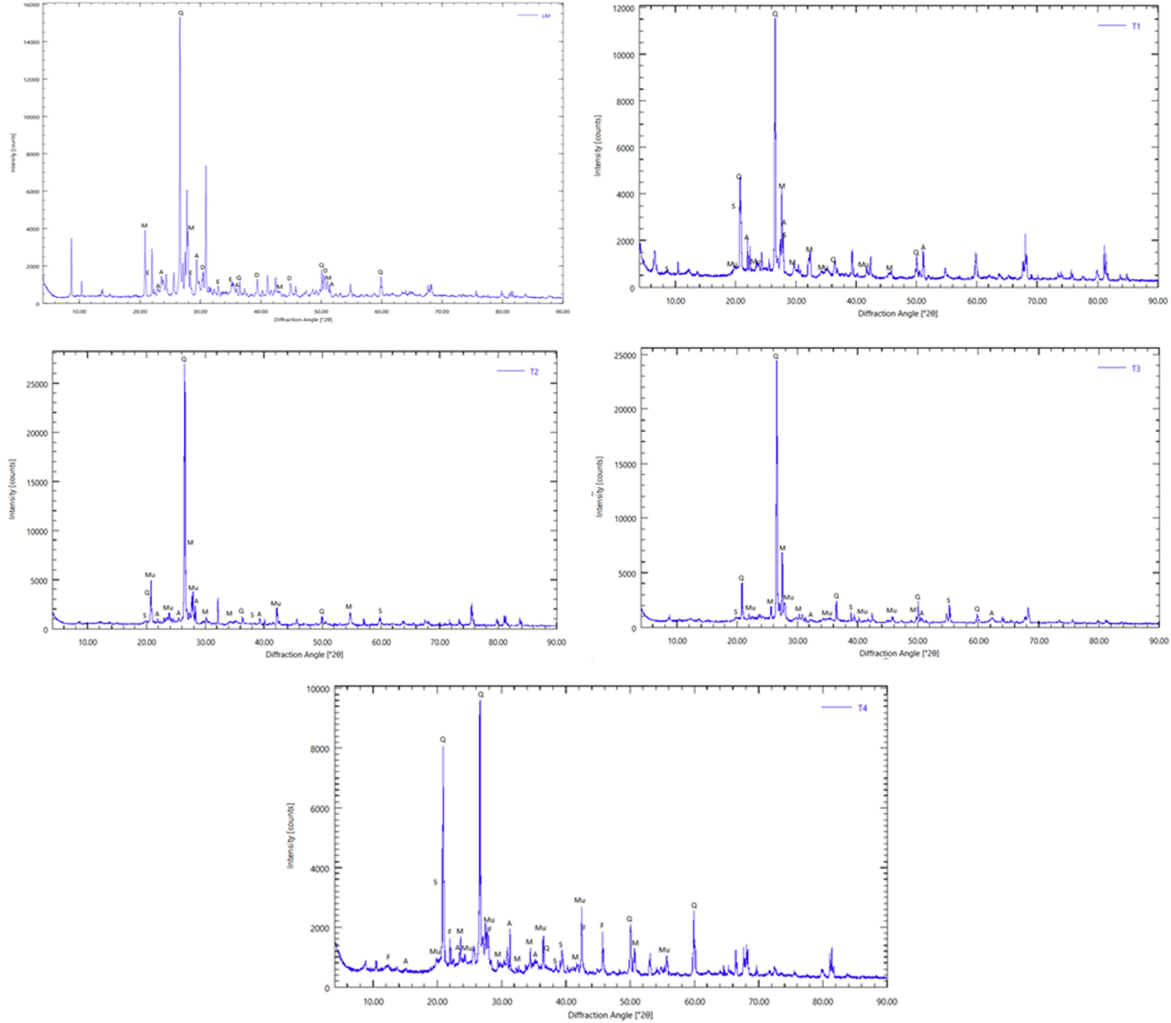
X-ray diffractogram. Q (Quartz), D (Dolomite), M (Microcline), A (Albite), E (Enstatite), Mu (Muscovite), S (Sepiolite), e F (Ferrosilite).

In the range of 18–30° 2θ, reflections attributed to the feldspars microcline and albite are observed, with closely spaced, partially overlapping peaks characteristic of feldspars. The simultaneous presence of K-feldspar (microcline) and Na-feldspar (albite) indicates a felsic composition typical of granitic or orthogneiss sources. The peaks around 30–31° 2θ correspond to dolomite, identified by Surendar et al.,^51^ as a carbonate accessory phase associated with sedimentary environments or hydrothermal alteration processes. This reflects secondary carbonate input, reactive under acidic conditions, releasing Ca and Mg. Smaller peaks in the region of 34–38° and 50–55° 2θ are compatible with enstatite (MgSiO_3_), an orthopyroxene representative of mafic or ultramafic components. Its detection, even if subtle, indicates heterogeneity, with minor ferromagnesian components materials in the analyzed stone waste.

Sharp quartz peaks confirm high crystallinity, while broader feldspar and dolomite peaks suggest microstresses or partial alteration also observed by Surendar et al.,^51^ in lateritic samples from the Pachamalai hills. The set of observed minerals (quartz, feldspars, dolomite, and enstatite) represents a mixed silicate-carbonate system derived from partially carbonated silico-aluminous rocks.

Dolomite exhibits chemical reactivity by releasing Ca^2+^ and Mg^2+^ in acidic environments. Quartz contributes mechanical strength, while feldspars weather to form secondary clays. As an agricultural input, stone waste mineralogy supports gradual release of Ca, Mg, and Si with high structural stability, favorable for soil remineralization. This mineralogical signature, according to the model proposed by Surendar et al.,^51^ suggests a complex geological origin and combines mechanical strength with moderate geochemical reactivity, which are desirable characteristics for technological and agricultural uses.

In the analysis of the diffractogram corresponding to treatment T1, classified as an Argisol, the soil was predominantly siliceous, with mica, feldspar, and clay fractions. Quartz dominated, with muscovite, feldspar, and sepiolite also present. These results are consistent with the findings of Surendar et al.,^51^ for soil samples from the Pachamalai Hills, in which feldspar and mica were also reported as the main mineral constituents.

Surendar et al.,^51^ also identified sepiolite in their samples, further supporting the compositional similarity between the studied soils. Additionally, Volvok et al.,^52^ reported that in silicate matrix soils, quartz, micas, and clays typically dominate the diffraction signal, and that detailed interpretation detailed interpretation requires complementary analytical methods, which corroborates the observations obtained in the present study.

From a pedological and geochemical perspective, feldspars and muscovite act as slow-release sources of potassium (K). Sepiolite and muscovite contribute to increasing the cation exchange capacity (CEC) and water retention of the soil. Quartz is a persistent mineral in the soil, whereas feldspars and micas undergo weathering to form secondary clay minerals, which, over time, can influence soil texture and fertility. The diffractogram of sample T2, which received the addition of 5% stone waste, indicated a showed a stronger, sharper quartz peak at 26–28° 2θ, suggesting a higher relative crystalline fraction of the silica mineral. Secondary peaks corresponding to muscovite and feldspar (microcline and albite) remained and became more distinct. In contrast, the sepiolite peaks were weak but detectable.

The addition of 5% rock powder enhanced the proportion of highly crystalline material detected in the analysis. Because X-ray diffraction (XRD) responds predominantly to crystalline phases, small quartz additions markedly increased peak intensity. This observation explains the significantly higher quartz peak observed in treatment T2.^52^

The addition of 5% stone waste increased the mineral fraction available for chemical reactions in the soil, including elements such as Ca, Si, Mg, and possibly Al. This enrichment may alter pH, nutrient availability, and OM adsorption. Surendar et al.,^51^ also demonstrated that rocks and sediments contain a wide range of minerals and elements that significantly affect surface chemistry. Therefore, the enhanced mineralogy observed in treatment T2 has practical implications for the geochemical behavior of soil.

Feldspars (microcline and albite) and muscovite act as sources of K, Na, and trace amounts of Si through weathering processes; however, the release of these elements occurs gradually over time. The presence of sepiolite, even in small amounts, together with muscovite, sepiolite and muscovite may enhance water retention and CEC. Over time, the weathering of muscovite and feldspars can lead to the formation of Fe and Al oxides, which are secondary clay minerals that contribute to increased cation exchange capacity and improved soil-water-holding capacity. The diffractogram of treatment T3, which received 10% stone waste, again shows a T3 showed dominant quartz, with visible mica and feldspar peaks, and weak clay signals. This is consistent with the typical mineralogy reported by Surendar et al.^51^ The lower baseline reflected dilution of amorphous phases by crystalline powder by the crystalline mineral powder. Volkov et al.,^52^ points out that organic fractions increase background noise in spectral techniques, whereas adding crystalline material improves the peak/background contrast.

The addition of 10% rock powder further increased the contribution of crystalline phases in the XRD pattern compared to samples without the addition, making the quartz and feldspar peaks more pronounced. This effect is expected, as crystalline materials, even in small amounts, strongly enhance diffraction peak intensity. The T3 treatment with 10% stone waste increased availability of Si, Ca, Mg, and Al, which may contribute to an increase in pH, cation exchange capacities, and slow nutrient release. Surendar et al.,^51^ also documents the elemental composition of the different rock powders, listing Si, Ca, Al, Fe, and others. The addition of mineral particles increases the surface area and adsorption sites for organic matter, which can alter organic C stabilization and aggregation properties. Volkov et al.,^52^ shows that organo-mineral associations affect spectroscopic signals and soil chemistry, as described in the FTIR results. The T4 treatment with the addition of 15% stone waste showed T4 dominant quartz with stronger secondary peaks (muscovite, feldspars, ferrosilite, sepiolite), with several identified as muscovite, feldspars (microcline and albite), ferrosilite, and sepiolite, many exhibiting clearly detectable intensities. The minor peaks in the medium and high ranges of 2θ were strengthened, indicating that the minor phases of the rock powder became detectable when their fraction increased to 15%.

The progressive addition of crystalline stone waste reduced organic matter signals in XRD, which increased the peak/background contrast and made smaller peaks detectable. With greater mineral addition, previously obscured reflections of muscovite, feldspars (albite and microcline), and sepiolite became detectable and appeared more distinct. The presence of more mineral surfaces increases adsorption sites for organic groups, altering the partitioning of organic matter between free and stabilized fractions, with consequences for carbon stability and aggregation properties. The T4 treatment is in line with the literature, as stone waste proves to be a source of quartz, feldspar, and muscovite. Its addition at 15% stone waste markedly altered soil mineralogy, with pedological implications discussed by Surendar et al.,^51^ and Volkov et al.^52^ The physical and chemical properties of the soil are altered, with a potential increase in cation exchange capacity, increased availability of Si and other cations (Ca, Mg, and Al) bound to the mineral, and an increase in pH values. The increase in mineral surface can promote the sorption and stabilization of organic matter (promoting physicochemical stabilization of organic matter), affecting the decomposition or retention of carbon. Trace nutrients such as Ca, Mg, K, and P in the rock powder may act as soil amendments, slowly releasing these elements. Monitoring for undesirable metals and radionuclides remains necessary (Table 6).

**Table 6:** Summary of Mineralogical and Functional Changes Across Treatments

treatment	stone waste	XRD characteristics	main minerals identified	key changes vs T1	pedological-geochemical implications
T1	0%	Baseline pattern; broader background	Quartz (dominant), muscovite, feldspar, sepiolite	Reference condition	Natural silicate soil; moderate CEC; OM influence on background signal
T2	5%	Increased peak intensity; sharper quartz peak (26–28° 2θ)	Quartz↑, muscovite, feldspar (microcline, albite), weak sepiolite	Increase in crystalline fraction	Initial enrichment in Ca, Mg, Si; slight pH increase; enhanced mineral reactivity
T3	10%	Further peak sharpening; reduced background noise	Quartz (dominant), clearer feldspar and mica peaks	Greater crystallinity; reduced amorphous influence	Increased availability of Ca, Mg, Si, Al; improved CEC; enhanced adsorption and organo-mineral interactions
T4	15%	Strongest peak/background contrast; minor phases more visible	Quartz (dominant), muscovite, feldspar, ferrosilite, sepiolite	Detection of minor phases; highest crystallinity	Greater nutrient reservoir (Ca, Mg, K, Si); increased pH and CEC; improved OM stabilization and aggregation potential

The addition of 10% stone waste (T3) further sharpened quartz and feldspar peaks in the XRD pattern, confirming greater crystallinity and reduced amorphous influence. This increased availability of Si, Ca, Mg, and Al contributed to higher pH, improved cation exchange capacity, and enhanced organo-mineral interactions. At 15% stone waste (T4), quartz remained dominant, but secondary minerals such as muscovite, feldspars, ferrosilite, and sepiolite became more visible, indicating that minor phases were detectable at higher doses. The progressive addition of crystalline material reduced organic matter signals in XRD, improving peak/background contrast and revealing smaller mineral reflections.

Soil degradation processes, widely documented in arid regions,^53^ highlight the need for sustainable and innovative soil management strategies. In this context, the use of mineral amendments, such as stone waste, represents a promising approach to restore soil fertility. The results obtained for treatments T2–T4 demonstrate that remineralization can enhance nutrient availability and organo-mineral interactions, supporting its applicability in tropical soils affected by degradation.

### 3.4 Surface Morphology and Surface Area

Scanning electron microscopy (SEM) was used to assess morphology and surface elemental composition. In this study, SEM was used to characterize the changes in soil morphology resulting from the addition of stone waste. Furthermore, with the aid of an energy-dispersive X-ray spectroscopy (EDS) detector, the chemical composition of each soil sample was analyzed, and the results provided comparative data for the XRD and XRF analyses. Figure 2 presents SEM images of stone waste samples (A) and of soil from Catalão treated with increasing doses of stone waste (T1­(B) [0%], T2­(C) [5%], T3­(D) [10%], and T4­(E) [15%]). The image in Figure 2A corresponds to stone waste; it shows stone waste particles were heterogeneous, angular, and rough, reflecting mechanical milling during beneficiation and processing. These characteristics can be seen in the studies by Hawerroth et al.^54^ de Oliveira et al.,^55^ and Wang, Yang, and Wu,.^56^ Evaluating images (B), (C), (D), and (E) from Figure 2, it is possible to notice some significant differences on the surface of the soil samples that received stone waste. As the stone waste dose increased, soil surface roughness also increased, likely linked to organic matter decomposition or complexation promoted by soil–stone waste interactions. Among these samples, T4 (15% SW) showed the most pronounced morphological changes.

**Figure 2:**
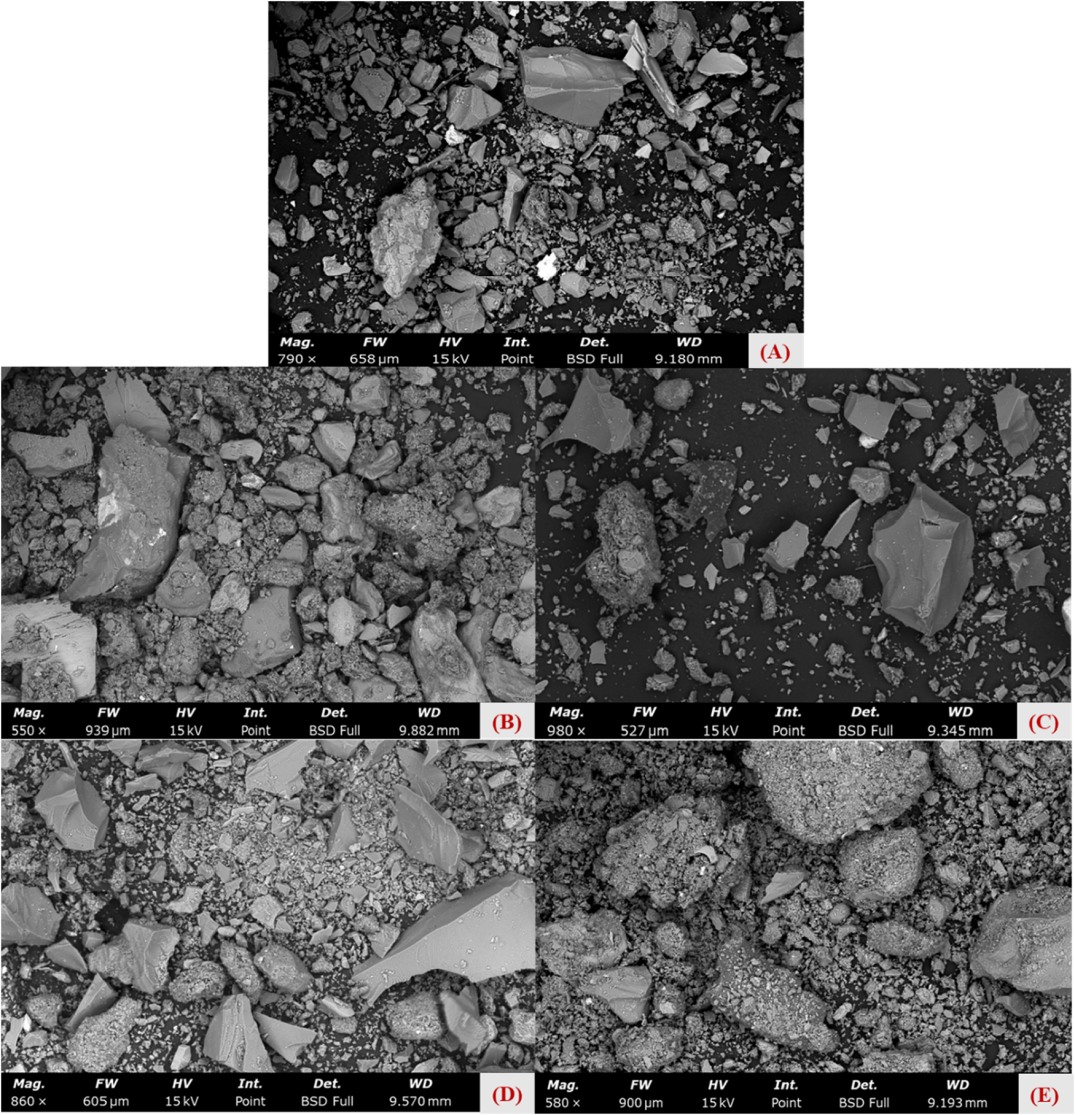
SEM do “raw” stone waste (A) e do solo do Catalão tratado com doses crescentes de stone waste (0% (B), 5% (C), 10% (D), 15% (E)).

EDS microanalysis (Figure 3) confirmed quartz, feldspars, and dolomite as dominant phases, in accordance with results previously determined by XRF, XRD, and FTIR. In treatment T1, Catalão soil without stone waste addition, contained C and H, consistent with high OM, a fact that corroborates the fertility analyses which found Corg contents above 8%. The sample also contained Si, Al, K, Ca, and Fe; the presence of these elements suggests the occurrence of silicate minerals such as feldspars, quartz, and clay minerals. In treatment T2 showed aluminosilicates and Ti-bearing minerals, with reduced OM. The absence of C indicates a reduction in OM content or complexation of OM. Treatment T3 contained O, Mg, Al, Si, P, K, Ca, Ti, and Fe. This T3 exhibited diverse phases including carbonates, silicates, and oxides, possibly associated with the various rock types present in the stone waste incorporated into the soil. In treatment T4, the elements found were O, Mg, Al, Si, P, K, Ca, and Fe, with a Treatment T4 resembled T3 but showed no detectable phosphorus. This suggests that higher doses of stone waste directly influence the soil elemental composition. The elemental variation across treatments confirmed remineralization effects.

**Figure 3:**
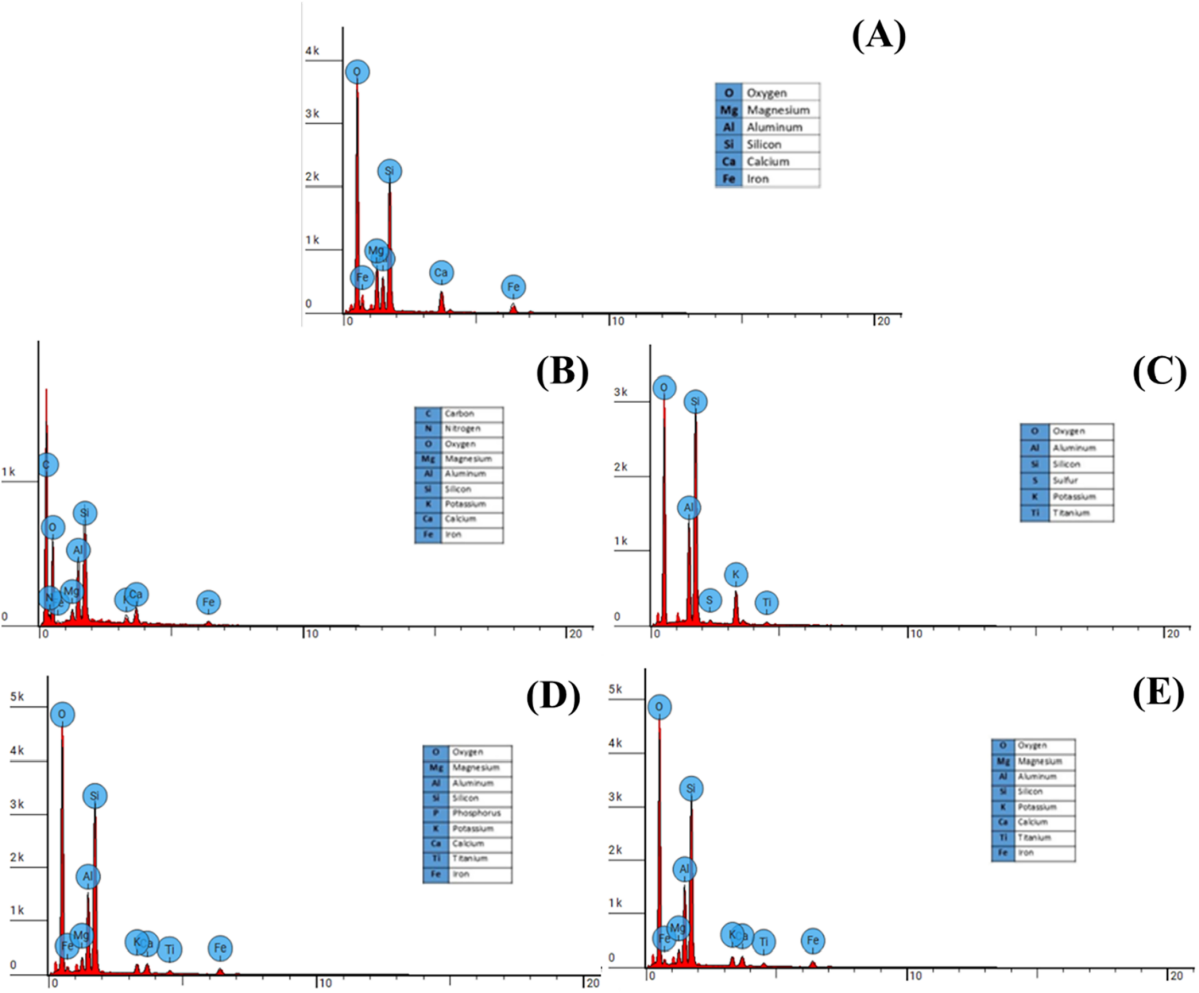
EDS do “raw” stone waste (A) e do solo do Catalão tratado com doses crescentes de stone waste (0% (B), 5% (C), 10% (D), 15% (E)).

Overall, the progression from T2 to T4 demonstrates that increasing stone waste doses enhance crystallinity, nutrient availability, and organo-mineral interactions, while modifying soil morphology and elemental composition. These changes support the potential of stone waste as a soil remineralizer but highlight the need for monitoring trace elements and radionuclides.

Stone waste contained diverse chemical elements but was dominated by O, Si, and Al (>70%), consistent with a granitic origin.^57^ This reflects that the material is a byproduct of granitic rocks mixed with other lithologies.^57^

### 3.5 Infrared Spectroscopy

Figure 4 shows the FTIR (Fourier Transform Infrared) spectra, which identifies functional groups. In the stone waste spectrum, bands at 2300–2000 cm^–1^ reflected adsorbed CO_2_ and carbonate release from pores. In studies with natural carbonates and silicates, these signals arise from both the environmental absorption of CO_2_ and the release of CO_2_ from surface carbonates.^51^ Nearby peaks indicated interactions with crystalline environments of Ca^2+^ and Mg^2+^.^52^ The peaks at 1433 and 877 cm^–1^ confirmed dolomite/carbonate phases, as already evidenced. The 1433 cm^–1^ peak, typical of dolomite (CaMg­(CO_3_)_2_), showed strong bands in the 1420–1470 cm^–1^ region. In the 1000–900 cm^–1^ region, a 992 cm^–1^ peak indicated Si–O stretching in feldspars and quartz, including albite and microcline such as albite, microcline, and quartz, corroborating the results of X-ray diffractometry. This indicates that the primary matrix was a tectosilicate. Volkov et al.,^52^ observed similar peaks in silica powders and granitic rocks. The 774 and 726 cm^–1^ peaks corresponded to microcline and albite, overlapping with quartz. Surendar et al.,^51^ describe equivalent bands in feldspathic minerals, reinforcing that the aluminosilicate fraction is dominant in the sample.

**Figure 4:**
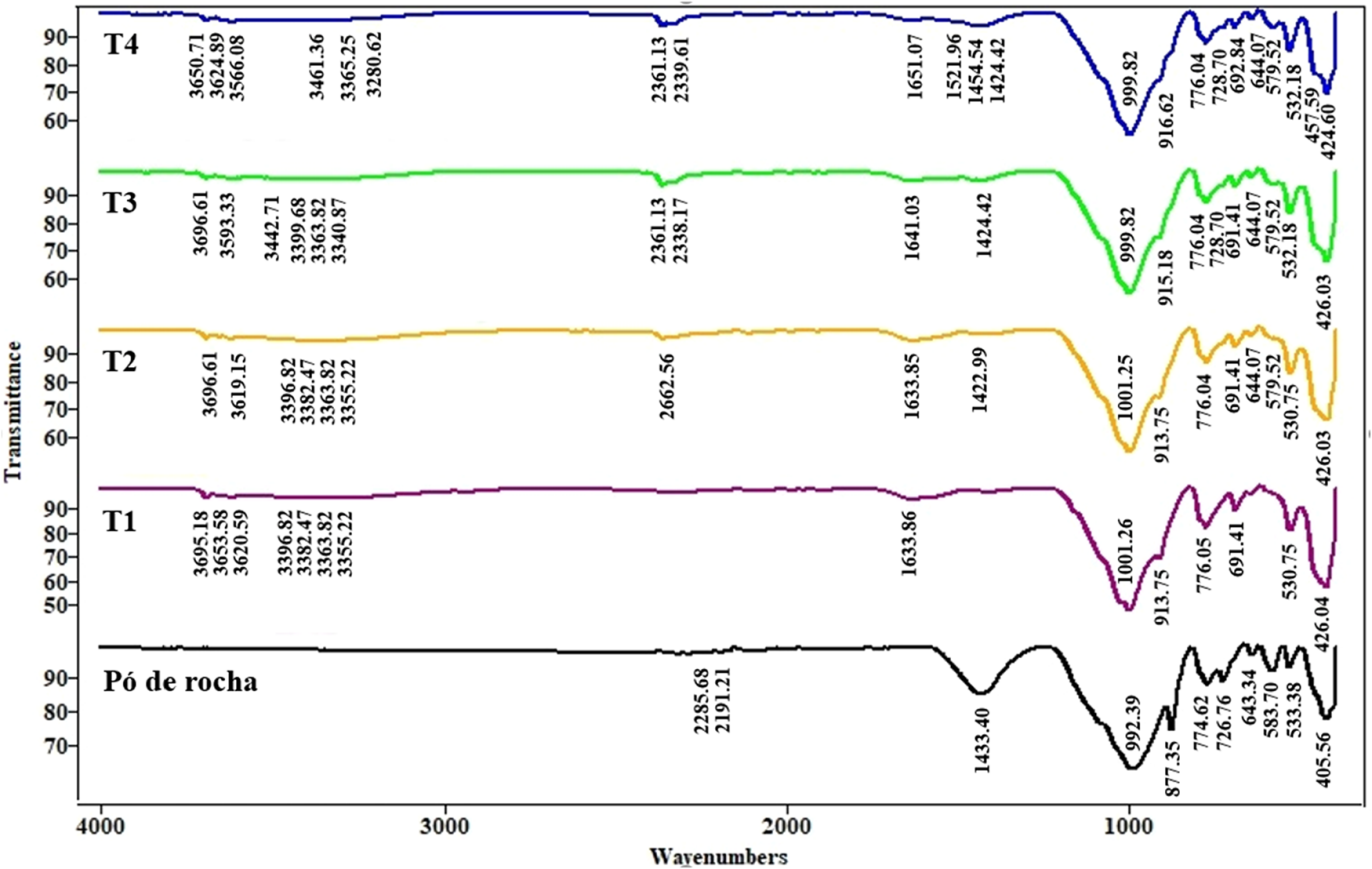
FTIR do stone waste and do solo do Catalão tratado com doses crescentes de stone waste, T1 (0%), T2 (5%), T3 (10%) and T4 (15%).

In the 400–650 cm^–1^ region, bands at 642–405 cm^–1^ reflected quartz, feldspar, and carbonate lattice vibrations with ionic substitutions (Ca/Mg). The natural rock powder samples showed numerous overlaps between carbonates, feldspars, and quartz, which is why the peaks may be broad and shifted. Isomorphic substitutions (Ca for Mg and Al for Si), crystal size, and contamination or moisture can slightly change these positions.

Regarding organic functional groups, the absence of OH and C=O bands indicated minimal free organic groups or highly free OH groups.

The reduction in organic-related FTIR signals after stone waste addition can be explained by two mechanisms: dilution and complexation/stabilization.

In the dilution effect, incorporation of mineral material (quartz, feldspars, carbonates), largely inorganic and crystalline, reduces the relative proportion of organic matter. As FTIR detects functional groups proportionally, this leads to a decrease in the intensity of organic bands.

In complexation and stabilization, mineral surfaces such as feldspars, carbonates, and secondary phases adsorb organic molecules through electrostatic interactions, cation bridging (Ca^2+^, Mg^2+^), and ligand exchange.

Together, these processes attenuate organic functional group signals in FTIR spectra. While dilution reduces signal intensity quantitatively, complexation modifies it qualitatively, reflecting increased stabilization of organic matter on mineral surfaces (Table 7).

**Table 7:** Summary of FTIR Changes across Treatments

treatment	stone waste	FTIR characteristics	organic bands	interpretation
T1	0%	Higher background; more evident organic-related signals	More detectable	Higher relative OM content; weaker mineral signal
T2	5%	Increased carbonate (1433 cm^–1^) and Si–O (∼992 cm^–1^) bands	Slightly reduced	Initial mineral enrichment; onset of OM dilution and interaction
T3	10%	Stronger silicate and carbonate bands; reduced overlap noise	Reduced	Increased crystallinity; enhanced organo-mineral interactions
T4	15%	Highest peak definition; dominant mineral bands	Minimal/weak	Strong dilution of OM; increased stabilization on mineral surfaces

Treatment T1 soil spectrum showed multiple defined peaks, consistent with diverse functional groups present. In the 3700–3600 cm^–1^ region, bands appear that are associated with the indicate kaolinite OH stretching; the 3620 cm^–1^ band corresponds to quartz.^51^ The broad 1100–1000 cm^–1^ band reflected Si–O stretching, with contributions from quartz, clays, and feldspars. In clay soil, this region usually shows a broad band with sub-bands attributable to the different tetrahedral environments of Al or Si. Surendar et al.,^51^ provides detailed tables of quartz and feldspar bands in this region, with the 1001 cm^–1^ band indicates H–O–H bending of water, characteristic of montmorillonite.

The peak at 913 cm^–1^ band indicates OH/Al–OH modes, consistent with smectite. The peaks between 776 and 695–678 cm^–1^ reflected Si–O vibrations in quartz and feldspars, as cited by Surendar et al.^51^ These bands help distinguish the relative contributions of quartz and feldspar. The peaks between bands at 650–400 cm^–1^ indicate clay, feldspar, and Fe oxide vibrations (goethite/hematite at ∼530 cm^–1^). Bands in this region are typical of clay minerals, iron oxides (goethite or hematite), and feldspar. This multiplicity of peaks indicates mineralogical mixing and isomorphic substitutions of Si with Al and Fe.^51^ The bands between 450 and 550 cm^–1^ and variations in intensity in the lower region are usually associated with goethite and hematite at 530 cm^–1^, according to Surendar et al.^51^ This confirmation is important because iron oxides control the soil color, cation exchange capacity (CEC), and nutrient adsorption. Broad 3400–3200 cm^–1^ OH bands and a 1633 cm^–1^ carbonyl/aromatic band confirmed humified organic matter, revealing carbonyl and aromatic groups characteristic of humification.^52^

In treatment T2 (5% stone waste), 1430–1415 cm^–1^ bands confirmed carbonate phases (dolomite, calcite) from stone waste (CO_3_^2–^) from stone waste. There was an Si–O band intensification reflected quartz/feldspar addition, reflecting the addition of silica- and feldspar-rich material present in stone waste. A possible shift in the maximum of this band may also occur due to the overlapping signals of feldspars and silica.^51^

The 913 cm^–1^ smectite band persisted after this treatment, representing the OH/Al–OH combination modes in clays, indicating the presence of smectite. New peaks at 800–700 and 600–400 cm^–1^ indicated feldspar and carbonate inputs (Si–O–Si, Al–O) and M–O vibrations (Ca–O, Mg–O), consistent with the presence of feldspar and carbonate derived from stone waste. Carbonate addition likely raised pH, supplying exchangeable Ca^2+^ and Mg^2+^. In acidic soils, this increases nutrient availability. Furthermore, the addition of silica and feldspar provides small amounts of Si, Al, K, and Na.^54^ Stone waste increased inorganic adsorption sites but did not replace clay CEC. This effect depends on the balance between the increase in inorganic sites and the dilution of clay.^54^ The addition of 5% rock powder resulted in an increase in the mineral bands (silicates, carbonates, and oxides). In the spectrum, this is manifested by the appearance of new inorganic bands (mainly in the 1000–400 cm^–1^ region) and modifications in the organic bands due to overlap and chemical interactions (complexation). Bands at 1650–1400 cm^–1^ indicated mineral–organic carboxylate complexes with Ca^2+^, Al^3+^, and Fe^3+^ released by rock powder. One of the agronomic implications of organic matter stability is the complexation between carboxylates and cations from stone waste, which tends to increase the physicochemical protection of OM. This explains why mineral–organic complexes stabilized organic matter, reducing soil mineralization due to greater chemical stability. Volkov et al.,^52^ discuss the strong contribution of water and overlap with silica proportions in this region.

The FTIR analysis of treatments T3 and T4 highlights how increasing stone waste doses alter soil mineralogy and organic matter interactions. At 10% stone waste (T3), carbonate bands intensified, Si–O peaks strengthened, and low-frequency bands indicated more carbonates and feldspars. This raised pH, increased Ca^2+^ and Mg^2+^ availability, and enhanced organo-mineral stabilization of organic matter. At 15% stone waste (T4), carbonate bands became dominant, Si–O peaks sharpened, and stronger M–O vibrations were observed. This improved base saturation but reduced immediate phosphorus availability due to precipitation and adsorption processes.

In the O–H band (3600–3200 cm^–1^), crystalline clays or oxides/hydroxides may be present; the rock powder can give rise to structural hydroxyls that appear as sharper peaks over a broad O–H envelope. Additionally, adsorbed water in the mineral powder can increase the bonded O–H component (broader and more intense band). In treatment T3, which received 10% stone waste, there was a 1430–1415 cm^–1^ carbonate bands intensified, reflecting dolomite input from the stone waste, thus making it easily detectable and with a higher relative intensity. A 992–1000 cm^–1^ Si–O band strengthened, consistent with quartz/feldspar addition, with an increase in the crystalline silica and feldspathic bands. The intensification of bands in the low-frequency region 650–400 cm^–1^ bands intensified, indicating carbonates and feldspars, reflect the increase in carbonates and feldspars contained in the stone waste. At 10% stone waste, pH increased and Ca^2+^ and Mg^2+^ availability rose, corroborated by fertility analyses. In acidic soils, this can reduce acidity and increase base availability, affecting P and micronutrients. The significant contributions of crystalline silica and feldspars can alter phosphorus sorption and provide plant-available Si in the long term. Clay peaks were slightly reduced, reflecting dilution by crystalline minerals. Clay sites continue to predominantly control ionic exchanges, although there may be a slight reduction in the nutrient retention capacity of colloids per unit mass of the total material. The addition of 10% stone waste may alter textural and physical properties, modifying density, porosity, and hydraulic conductivity, depending on particle size distribution.^54^ With the application of 10% stone waste, it is expected that the Si–O and M–O bands will increase in intensity, precisely where this spectrum shows distinct peaks confirmed silicate, carbonate, and oxide contributions. This confirms the direct contribution of the added mineral—silicates, carbonates, and oxides. Bands at 1600–1550 and 1450–1400 cm^–1^ indicated organic–mineral carboxylates, stabilizing soil organic matter.

In treatment T4, with the addition at 15% stone waste, the carbonate band at 1430 cm^–1^ became dominant (CO_3_^2–^). There was a Si–O peak shifted to 990–1000 cm^–1^, reflecting quartz/feldspar enrichment.

The 1630 cm^–1^ OH band intensified, suggesting larger surface area or residual clays. Bands at 650–400 cm^–1^ increased, indicating stronger Ca–O, Mg–O, and Al–O vibrations, suggesting an increase in Ca–O, Mg–O, and Al–O vibrations associated with carbonates and feldspars. The 15% stone waste treatment produced stronger pH correction due to carbonate input, directly influencing phosphorus availability. Under these conditions, phosphorus can be immobilized through precipitation reactions, particularly as Ca-phosphates, as well as through enhanced adsorption onto mineral surfaces such as carbonates, silicates, and Fe/Al oxides. Consequently, although the addition of stone waste improves base saturation through Ca^2+^ and Mg^2+^ inputs, it may reduce the immediate availability of P due to these precipitation and adsorption processes at higher pH. The finer particle size of stone waste promotes a faster reaction. Ideally, pH should be monitored over time. At 15% stone waste, soil texture and matrix properties were altered, with changes in porosity and density, influencing infiltration and retention. When we evaluate the effect of applying 15% stone waste, there is stronger carboxylate complexation observed, as the changes in the 1650–1400 cm^–1^ ranges suggest greater formation of coordinated carboxylates (−COO^–^ binding Ca^2+^/Al^3+^/Fe^3+^ released by stone waste). This indicates that free −COOH groups were converted into complexed carboxylates. The appearance of the peak at 1430 cm^–1^ suggests the presence of carbonate, which alters local pH and complexation. As stone waste concentration increased, organic bands (O–H, C–H, C=O) decreased, indicating organic matter–mineral interactions, suggesting the interaction of organic matter with the mineral material. Thus, the most reactive minerals in stone waste, such as Fe, Al, Si, Mg, and Ca complexed with humic compounds, adsorbing OM, making them harder to detect by FTIR. Additionally, the 1000–1100 cm^–1^ Si–O peak sharpened, reflecting enrichment in silicates, feldspars, and metallic oxides from stone waste. effects were strongest in T4.

Overall, treatments T3 and T4 demonstrate that higher stone waste doses enhance mineral contributions, improve base saturation, and stabilize organic matter, but may reduce phosphorus availability at elevated pH.

### 3.6 Influence of Stone Waste on the Physical Properties Density and Porosity

Soil structure governs water dynamics, root growth, biota, and aeration. In this context, the attributes soil density (Ds) and porosity (total, macro, and micro) serve as indicators of the impact that different stone waste treatments had on soil structure (Table 8). The behavior of the Catalão soil studied here was atypical, because even though it is classified as a Red-Yellow Argisol, its high OM (7–8%) in the A horizon reduced density and increased porosity. of the studied soil. Since this A horizon had a density of 0.73 g cm^–3^. The stone waste raised density to 1.03 (T2), 0.89 (T3), and 0.93 g cm^–3^ (T4). Soil density (g cm^–3^) reflects compaction and overall porosity. The stone waste increased density initially; weathering and aggregation may reduce it over time. Additionally, intensified biological cycling driven by nutrient release can compensate for this initial effect, stimulating greater biogenic porosity. Macropores (diameter >50 μm) are responsible for aeration, infiltration, and redistribution of water in the soil profile. The addition of stone waste, due to its very fine particles, initially tends to fill larger spaces between aggregates, reducing the continuity and volume of macroporosity fell from 18% (T1) to 8–12% (T2–T4). T2, T3, and T4, respectively. However, in the medium and long-term, the interaction between minerals released by weathering and biological activity (roots, microorganisms, exudates) can favor the formation of new stable aggregates, restoring some of the lost macroporosity. Microporosity rose in T2 (35%) but declined in T3 (29%) and T4 (26%) in micropores, and these reductions linked to OM decomposition after stone waste incorporation

**Table 8:** Physical Parameters

origin	particle density (g cm^–3^)	total porosity (%)	Ds (g cm^–3^)	macropores (%)	micropores (%)
Stone Waste	2.706	Nd	Nd	Nd	Nd
Catalão Soil (T1)	2.528	51	0.73	18	34
T2	2.539	43	1.03	8	35
T3	2.541	41	0.89	12	29
T4	2.555	38	0.93	12	26

The density of the particles (g cm^–3^), an intrinsic property of the solid constituents, depends on the mineralogy of the material. Stone waste, generally composed of silicates (such as feldspars, orthopyroxenes, quartz, and micas), has a stone waste mineral density ranged 2.6–3.0 g cm^–3^ g cm^–3^, higher than that of organic matter (<0.8 g cm^–3^). Thus, its incorporation into soil tends to increase particle density, especially in organic or highly weathered soils, where the mineral fraction has less diversity. In this way, the particle density was 2.706 (SW), 2.528 (T1), 2.539 (T2), 2.541 (T3), 2.555 (T4). Therefore, the addition of stone waste to the soil may result in a stone waste caused temporary macroporosity loss, microporosity gain, initial bulk density rise, and particle density increase because of the mineralogy of the material added. These effects are dynamic and should be assessed according to exposure time, interaction with organic matter, and the biological activity of the soil. Thus, the application of stone waste should not be considered only as a chemical conditioner (nutrient release), but also as a potential agent of physical modifications, which can directly impact water dynamics, aeration, and root development.

### 3.7 Statistical Analysis

Principal Component Analysis PCA assessed relationships among soil physicochemical variables under SW treatments and the effects of different proportions of stone waste applied (T1 = 0%, T2 = 5%, T3 = 10%, and T4 = 15%). The PC1 explained 65.8% variance; PC2 26.0%; cumulative 91.9%. These results indicate that the two-dimensional PCA projection captured most variance of the multivariate relationships among the variables within the soil–stone waste system (Figure 5).

**Figure 5:**
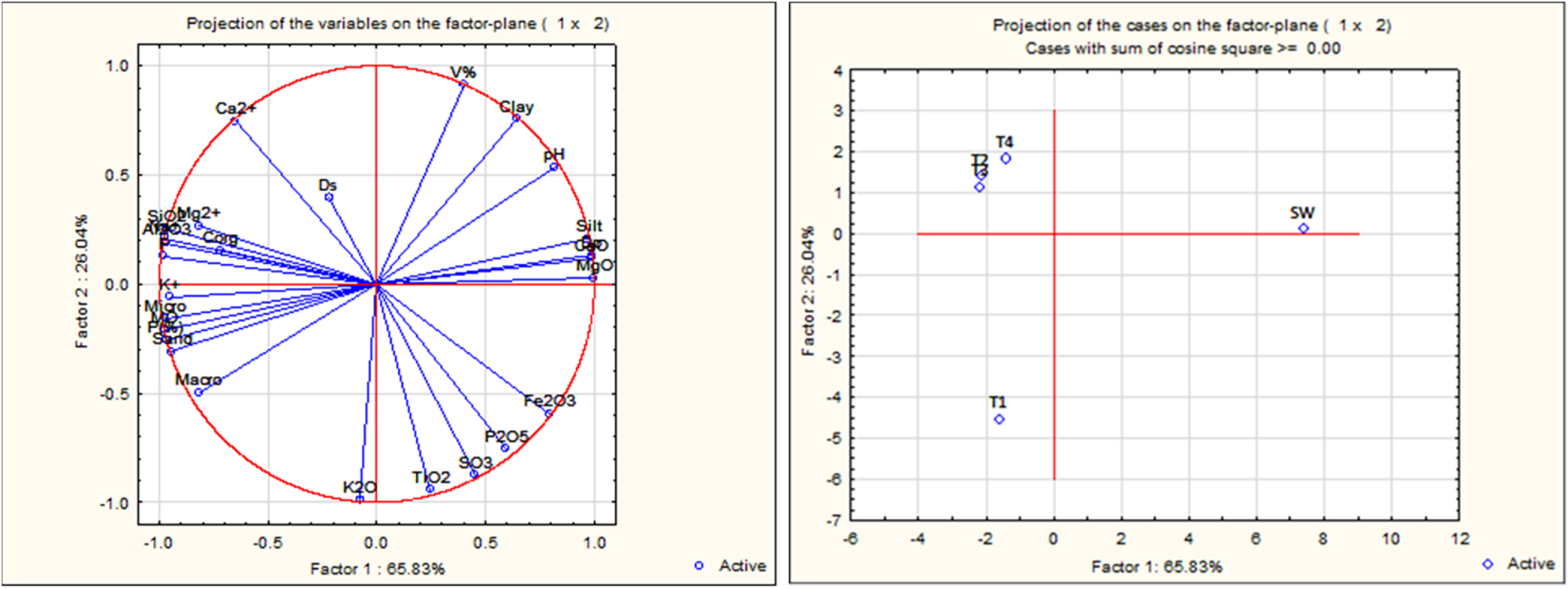
Principal component analysis (PCA).

Factor 1 (65.83%) separates the variables associated with soil fertility and the elemental chemical composition derived from stone waste, showing positive correlations with Fe_2_O_3_, P_2_O_5_, SO_3_, TiO_2_, and K_2_O, and negative correlations with SiO_2_, Al_2_O_3_, and Mg^2+^. This distribution indicates that the increase in stone waste dosage, particularly in treatments T3 and T4, is positively related to the enrichment in oxides and macronutrients derived from the rock, such as phosphorus (P_2_O_5_), potassium (K_2_O), and sulfur (SO_3_), as well as elements like iron and titanium, which are typical of silicate minerals and oxides present in mafic rocks. Conversely, variables such as SiO_2_, Al_2_O_3_, Mg^2+^, and Corg exhibited opposite vectors (negative side of F1), indicating that T1 (soil without rock powder) retains chemical characteristics dominated by the original soil matrix, with higher silica and aluminum contents and a lower contribution from basic minerals.

Factor 2 (26.04%) is primarily associated with the physical and structural properties of the soil, showing prominent vectors for Ca^2+^, base saturation (V%), clay content, and pH, all positioned positively along this axis. These parameters indicate an improvement in surface reactivity and cation exchange capacity (CEC) with increasing stone waste doses, reflecting the corrective and structural effects of the remineralizing material on the soil system.

The grouping of Corg, K^+^, Micropores, and Sand near the origin indicates a low influence of the stone waste doses on these attributes in the short term, which is consistent with the slow nature of nutrient release from ground rocks. The clear separation of chemical variables in the PCA shows that the addition of stone waste significantly altered the geochemical composition and potential fertility of the soil, especially from the 10% application rate onward. The increase in Ca^2+^, pH, and V% suggests improved surface reactivity and reduced soil acidity, effects that are compatible with the use of basaltic or syenitic rock remineralizers. Additionally, the enrichment in Fe_2_O_3_ and TiO_2_ reflects the incorporation of mafic minerals (such as ilmenite and magnetite), which can serve as long-term sources of micronutrients. The correlation between P_2_O_5_ and K_2_O reinforces the potential of stone waste as a complementary source of phosphorus and potassium for weathered tropical soils, such as Argisols. The projection of the treatments (Stone waste, T1, T2, T3, and T4) on the plane formed by the first two principal components (F1 and F2) explains 91.87% of the total variance in the data (F1 = 65.83% and F2 = 26.04%), ensuring a high representativeness of the multivariate structure of the soil–stone waste system.

Stone waste (pure rock powder) is positioned at the positive extreme of the F1 axis, indicating a strong correlation with chemical variables associated with the rock’s mineral content, such as Fe_2_O_3_, TiO_2_, P_2_O_5_, K_2_O, and SO_3_. This position shows that the remineralizing material has some geochemical profile rich in metallic oxides and slow-release nutrients. Treatment T1 (soil with 8% organic matter, without rock powder addition) appears at the negative extreme of F1, reflecting a composition dominated by SiO_2_, Al_2_O_3_, Mg^2+^, and Corg, which are typical of the original matrix of the Argisol, characterized by strong weathering and low levels of exchangeable bases. The isolated position of T1 demonstrates the chemical distance between the natural soil and the samples that received the remineralizer. Meanwhile, treatments T2, T3, and T4 are located between stone waste and T1, showing a gradient of progressive mineral enrichment with increasing doses of rock powder. T2 (5%) occupies an intermediate position, closer to T3 (10%), and T4 (15%) appears grouped in the upper left quadrant, close to each other, indicating compositional similarity and correlated chemical behavior; in other words, from 5% application onward, the effects of stone waste on the soil become consistent and significant. The arrangement of the points shows a gradual transition between untreated soil and stone waste, indicating effective interaction between both materials. Treatments with 10 and 15% stone waste (T3 and T4) approach the chemical profile of stone waste, suggesting a higher level of remineralization, characterized by increased levels of Fe_2_O_3_, P_2_O_5_, TiO_2_, and K_2_O, higher pH and base saturation (V%), reduced exchangeable acidity, and improved nutrient availability. These findings support the hypothesis that rock powder acts as an effective remineralizer, gradually increasing soil fertility and promoting chemical transformations that can benefit plant growth in the medium and long-term.

The Pearson correlation analysis revealed consistent relationships among the chemical, physical, and mineralogical attributes of the studied system, indicating well-defined geochemical patterns (Table 9).

**Table 9:** Pearson Correlation Analysis

	SiO_2_	CaO	Al_2_O_3_	K_2_O	MgO	Fe_2_O_3_	TiO_2_	P_2_O_5_	SO_3_	pH	P	K^+^	Ca^2+^	Mg^2+^	Na^+^	V%
SiO_2_	1	–0.92	0.98	–0.17	–0.95	–0.91	–046	–0.76	–0.65	–0.65	0.97	0.90	0.81	0.86	0.99	–0.15
CaO	–0.92	1	–0.94	–0.18	0.98	0.72	0.14	0.48	0.35	0.88	–0.963	–0.96	–0.55	–0.78	–0.94	0.50
Al_2_O_3_	0.98	–0.94	1	–0.13	–0.94	–0.89	–0.44	–0.67	–0.62	–0.71	0.99	0.94	0.75	0.88	0.99	–0.20
K_2_O	–0.17	–0.18	–0.13	1	–0.11	0.54	0.92	0.67	0.84	–0.58	–0.07	0.09	–0.67	–0.25	–0.14	–0.94
MgO	–0.95	0.98	–0.94	–0.11	1	0.76	0.20	0.59	041	0.82	–0.95	–0.93	–0.63	–0.79	–0.95	0.42
Fe_2_O_3_	–0.91	0.72	–0.89	0.54	0.76	1	0.76	0.89	0.88	0.33	–0.87	–0.75	–0.94	–0.86	–0.90	–0.23
TiO_2_	–0.46	0.14	–0.44	0.92	0.20	0.76	1	0.81	0.97	–0.25	–0.38	–0.18	–0.86	–0.42	–0.44	–0.76
P_2_O_5_	–0.76	0.48	–0.67	0.67	0.59	0.89	0.81	1	0.89	0.04	–0.63	–045	–0.97	–0.61	–0.71	–0.43
SO_3_	0.65	0.35	–062	0.84	041	0.88	0.97	0.89	1	–0.06	–0.57	–0.39	–0.94	–0.58	–0.63	–0.62
pH	–0.65	0.88	–0.71	–0.58	0.82	0.33	–0.25	0.04	–0.06	1	–0.75	–0.81	–0.14	–0.49	–0.69	0.81
P	0.97	–0.96	0.99	–0.07	–0.95	–0.87	–0.38	–0.63	–0.57	–0.75	1	0.96	0.71	0.88	0.99	–0.26
K^+^	0.90	–0.96	0.94	0.09	–0.93	–0.75	–0.18	–045	–0.39	–0.81	0.96	1	0.52	0.90	0.93	–0.41
Ca^2+^	0.81	–0.55	0.75	–0.67	–0.63	–0.94	–0.86	–0.97	–0.94	–0.14	0.71	0.52	1	0.65	0.77	0.41
Mg^2+^	0.86	–0.78	0.88	–0.25	–0.79	–0.86	–0.42	–0.61	–0.58	–0.49	0.88	0.90	0.65	1	0.88	–0.06
Na^+^	0.99	–0.94	0.99	–0.14	–0.95	–0.90	–0.44	–0.71	–0.63	–0.69	0.99	0.93	0.77	0.88	1	–0.19
V%	–0.15	050	–0.20	–0.94	0.42	–0.23	–0.76	–0.43	–0.62	0.81	–0.26	–0.47	0.41	–0.06	–0.19	1
MO	0.89	–0.98	0.89	0.28	–0.98	–0.64	–0.02	–0.44	–0.24	–0.89	0.91	0.92	0.48	0.73	0.9	–0.58
Corg	0.89	–0.98	0.89	0.28	–0.98	–0.64	–0.02	–0.44	–0.24	–0.89	0.91	0.92	0.48	0.73	0.9	–0.58

Strong positive correlations were observed among SiO_2_, Al_2_O_3_, and Na^+^ (*r* > 0.95), suggesting a common origin associated with aluminosilicate minerals. In contrast, CaO and MgO showed strong positive correlations with each other (*r* ≈ 0.99) and negative correlations with silicatic components, reflecting the influence of basic or carbonate phases.

The significant negative correlation between CaO and P (%) (*r* ≈ −0.99) indicates that phosphorus availability may be controlled by precipitation reactions with calcium, a mechanism widely reported in alkaline or Ca-rich systems. According to Lindsay, 1979,^59^ phosphorus tends to form low-solubility calcium phosphates under such conditions, reducing its mobility and bioavailability. Similarly, the positive correlation between pH and CaO reinforces the role of exchangeable bases in regulating soil alkalinity.

Organic matter (MO) and organic carbon (Corg) were strongly correlated (*r* ≈ 0.99), confirming data consistency, and also showed positive associations with P (%), suggesting a contribution of organic fractions to nutrient retention and cycling. This behavior is supported by Brady and Weil,^60^ who highlight the role of soil organic matter in improving nutrient availability and soil structure.

## 4 Conclusions

Overall, the results demonstrate that the application of ornamental stone waste contributes to improving soil fertility and mineralogical balance, supporting its role as a sustainable soil amendment. These findings align with current frameworks of sustainable land management aimed at mitigating land degradation and promoting long-term soil resilience.^61^ Ornamental stone waste showed significant potential as a physical and chemical soil conditioner, improving soil structure and fertility. Incorporating 5% stone waste increased the mineral fraction available for soil reactions, enhancing Ca, Mg, and Si, and promoting changes in mineralogy and structure. X-ray diffraction revealed intensified quartz peaks and sharper feldspar and mica signals, indicating increased crystallinity and potential effects on cation exchange capacity and water retention. Physically, stone waste modified pore distribution, temporarily reducing macroporosity while increasing microporosity, improving water retention in sandy soils. In the medium to long-term, interactions between weathered minerals and biological activity may favor new aggregate formation, partially restoring soil structure. Overall, ornamental stone waste should be considered not only a slow-release nutrient source but also a structural amendment influencing water dynamics, aeration, and root development. Thus, applying ornamental stone waste offers a promising sustainable strategy for Brazilian agriculture, reducing dependence on imported fertilizers and promoting environmentally responsible reuse of mineral industry residues.
